# The complete mitochondrial genome and phylogeny of *Diacronema viridis* (Pavlovales, Pavlovophyceae)

**DOI:** 10.1080/23802359.2021.1915713

**Published:** 2021-05-21

**Authors:** Su Yeon Kim, Sunju Kim, Eun Chan Yang

**Affiliations:** a Korea Inter-University Institute of Ocean Science, Pukyong National University, Busan, Republic of Korea; b Department of Oceanography, Pukyong National University, Busan, Republic of Korea; c Division of Earth Environmental System Science, Pukyong National University, Busan, Republic of Korea; d Marine Ecosystem Research Center, Korea Institute of Ocean Science & Technology, Busan, Republic of Korea; e Department of Ocean Science, University of Science and Technology, Daejeon, Republic of Korea

**Keywords:** *Diacronema viridis* CCMP 620, haptophytes, mitochondrial genome, Pavlovophyceae

## Abstract

The complete mitochondrial genome of the pavlovophycean microalga *Diacronema viridis* CCMP 620 was sequenced and characterized. The circular mitogenome is a total 29,282 bp in length with 39.2% GC content and contains 47 genes, including 20 protein-coding, three rRNA, and 24 tRNA genes. The gene synteny of *D. viridis* and *D. lutheri* has been highly conserved; however, the gene content (absence of introns and ORFs) and repeat regions (3.7 kbp) of *D. viridis* contributed to significant difference of mitogenomes within the *Diacronema*.

*Diacronema viridis* (C.K.Tseng, Chen & Zhang) Bendif & Véron is a member of pavlovophycean microalga (Haptophytes) that is one of the important primary producer in coastal ecosystems. Recent ultrastructure, pigmentation, and molecular phylogeny supported the uniqueness and monophyly of the genus *Diacronema*. Six species of *Diacronema* shared by the unique structure of the posterior flagellum with a basal swelling and vestigial (Bendif et al. 2011), and a typical haptophyte simple pigment type A comprises chlorophylls a, c1, c2, and Mg-Divinyl protochlorophyllide and carotenoids fucoxanthin, diadinoxanthin, diatoxanthin, and β-carotene (Van Lenning et al. 2003). Nuclear ribosomal RNA (18S and 28S rRNAs) phylogenies suggested relationships of six verified species and also genetic diversity to be uncovered in the genus. In the present study, we reported the complete mitochondrion genome (mtDNA) of *Diacronema viridis* CCMP 620, which is the second mitogenome of the genus.

Total genomic DNA was extracted from culture strain CCMP 620 denoted as unidentified *Pavlova* sp. (https://ncma.bigelow.org) by using a NucleoBond AXG 100 Kit (Macherey-Nagel GmbH & Co. KG, Düren, Germany). The identity of the strain was confirmed by using standard sequencing. Nuclear 18S rRNA sequence of the strain CCMP 620 was identical (100% identity) to that of *Diacronema viridis* KMMCC H-13 (HQ877913; Hur et al. 2015). The Truseq Nano DNA Preparation Kit (550 bp average insert size) was used for the NGS library construction and followed by whole genome sequencing using Illumina Hiseq 2500 (101 bp paired-end; Macrogen, Seoul, Korea). A mitochondrial contig was assembled with the NOVOPlasty (Dierckxsens et al. 2017). The raw reads were mapped on the contig to confirm the circular mtDNA sequences using the CLC Genomics Workbench ver. 6.5.1 (https://digitalinsights.qiagen.com). The complete mtDNA was recovered with 876.5 average coverage (minimum 144X, maximum 3,826X). Annotation was done with program Geneious^®^ ver. 11.1.5 (www.geneious.com) assisted by a *Diacronema lutheri* (MN564259, Hulatt et al. 2020), tRNAscan-SE On-line (Lowe and Chan 2016), and NCBI BLAST searches (www.blast.ncbi.nlm.nih.gov).

New mtDNA of *D. viridis* CCMP 620 (MW044629) is 29,282 bp in length, has a 39.2% GC content, encodes 47 genes (22,294 bp; 76.2% of mtDNA) including 20 protein-coding sequences, three rRNAs and 24 tRNAs. The mtDNA (15 CDS combined data) phylogeny supported the monophyly of haptophytes (100% bootstrap support) and *Diacronema* (Pavlovophyceae; 100%) (Figure 1). We compared the new mtDNA to that of *D. lutheri* NIVA-4/92, i.e. 36,202 bp complete mtDNA (MN564259, Hulatt et al. 2020). The gene synteny was highly conserved and identities of CDSs of *D. viridis* CCMP 620 to *D. lutheri* NIVA-4/92 ranged from 66.4% (rps14) to 87.7% (atp9). The cox3 of *D. viridis* is only CDS comprising of two exons (764 bp and 61 bp) with an intron (839 bp) which has similar structure with that of *D. lutheri*, two exons with an intron (831 bp). All the other 19 CDS and three rRNAs of *D. viridis* have no introns, cf. the atp6, atp9, cox1 and rnl and rns rRNAs including introns in *D. lutheri*. Two ORFs of *D. lutheri* (orf105 and cox1-intronic orf636) are absent in *D. viridis*. Identities of the tRNAs ranged from 81.1% (tRNA-Arg) to 100% (tRNA-Gly), and those of rnl and rns rRNAs were 79.7% and 85.9%, respectively. Five repeat units (total 3,764 bp) found between cox3 and rrl rRNA, i.e. two simple repeats (350 bp and 349 bp for repeat unit-1; 132 bp for repeat-4), one tandem repeat (2 × 85 bp and 84 bp for repeat-3) and two inverted repeats (79 bp for IR-1; 559 bp and 549 bp for IR-2). Complicated repeat regions between the genes are congruent to the mtDNAs of *Diacronema* such as 5.4 kbp for *D. lutheri* NIVA-4/92 and the longer than 2.7 kbp for *D. lutheri* CCMP 1325 (HQ908424; partial). The new mtDNA data would contribute to the conservation and industrial application of *Diacronema* as marine genetic resources.

**Figure 1.**
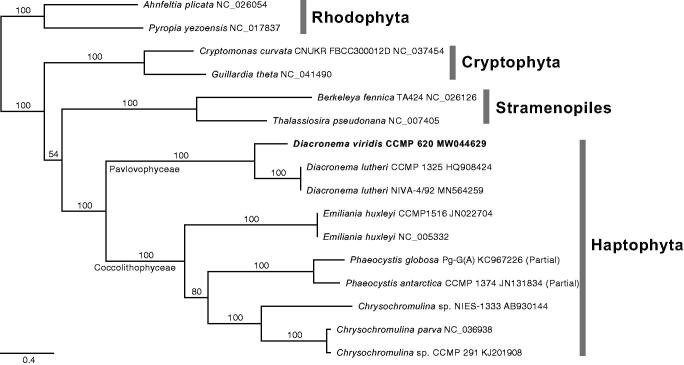
The maximum-likelihood mtDNA phylogeny of haptophytes based on 15 combined CDS data, including 10 haptophytes and six outgroups (Cryptophytes, Rhodophyta, and Stramenopiles). All CDS were aligned with translated amino acids and then concatenated as a total of 13,254 positions without ambiguous regions. The best phylogeny inferred under the GTR with independent heterogeneity (GTR + G) model for total 45 partitions, i.e. three codon positions of each gene. Bootstrap support values were calculated with 1,000 replicates using the same substitution model. The best phylogeny supports sister relationship of *Diacronema viridis* and *D. lutheri* with maximum bootstrap value.

## Data Availability

Newly determined mitogenome is available in on-line at https://www.ncbi.nlm.nih.gov under the accession no. MW044629. The associated BioProject, SRA, and Bio-Sample numbers are PRJNA691634, SRP301484, and SAMN17301202, respectively.
